# Cigarette Brand Use and Sexual Orientation: Intersections With Gender and Race or Ethnicity

**DOI:** 10.5888/pcd18.210160

**Published:** 2021-10-28

**Authors:** Alexandra Budenz, Rachel Grana

**Affiliations:** 1 National Cancer Institute, Tobacco Control Research Branch, Behavioral Research Program, Division of Cancer Control and Population Sciences, Rockville, Maryland

## Abstract

**Introduction:**

Lesbian, gay, and bisexual (LGB) populations have higher cigarette smoking rates than heterosexual populations. The tobacco industry has leveraged LGB, gender, and racial or ethnic identities to establish cigarette brand preference. We examined cigarette brand use among smokers by sexual orientation and the implications of gender and race or ethnicity for brand use.

**Methods:**

We used the National Survey on Drug Use and Health (NSDUH; 2015–2017) to conduct weighted bivariate analyses in 2019–2020 of the prevalence of 5 commonly used cigarette brands among adult smokers (N = 24,310) by sexual orientation. We conducted weighted regressions to test relationships between sexual orientation and brand use and interactions between sexual orientation, gender (defined in NSDUH as male or female), and race or ethnicity.

**Results:**

LGB smokers were more likely to use Camel (lesbian/gay, OR = 1.7 [95% CI, 1.2–2.3], bisexual, OR = 1.8 [95% CI, 1.5–2.2]) and American Spirit cigarettes (lesbian/gay, OR = 2.8 [95% CI, 1.9–4.1], bisexual, OR = 3.2 [95% CI, 2.5–4.1]) than heterosexual smokers. Lesbian/gay smokers had higher odds of Marlboro cigarette use (OR = 1.2; 95% CI, 1.0–1.4) than heterosexual smokers. Bisexual smokers were more likely to smoke Newport cigarettes (OR = 1.7; 95% CI, 1.4–2.1) than heterosexual smokers. Interactions between LGB and female identities (vs gay or bisexual male) were positively associated with Camel, Marlboro, and Newport use. The interaction between lesbian/gay and Hispanic/Latino ethnicity (vs lesbian/gay White) was also positively associated with Newport use.

**Conclusion:**

LGB smokers may be more likely to smoke some commonly used cigarette brands than heterosexual smokers, and gender and race or ethnicity may have implications for brand preference. Future research could examine specific contributors to brand use among LGB smokers (eg, tobacco marketing).

**Summary**

**What is already known about this topic?**

Little is known about cigarette brand preference (which encourages sustained smoking behavior) among lesbian, gay, and bisexual (LGB) smokers, who have higher cigarette smoking rates than heterosexual populations.

**What is added by this report?**

We conducted an intersectional analysis of brand use in LGB smokers, considering the roles of gender and race or ethnicity. This study also highlights the importance of studying LGB population subgroups, because we found differences not only in brand use between LGB and heterosexual smokers but also among LGB subgroups.

**What are the implications for public health practice?**

Understanding brand preference among LGB smokers may inform tobacco regulation at the federal, state, and local levels pertaining to LGB-targeted marketing of specific cigarette brands.

## Introduction

Cigarette smoking is the leading preventable cause of death in the US, and studies show that lesbian, gay, and bisexual (LGB) populations have higher cigarette smoking rates than the general population (20%–27% vs 14%) (1,2). One contributor to smoking disparities among LGB populations is targeted tobacco industry marketing. Starting more than 3 decades ago, the tobacco industry was one of the first commercial industries to target its marketing to LGB populations (3–5). Accordingly, LGB populations are more likely to be exposed to tobacco-related media and may be more receptive to tobacco marketing than heterosexual populations (6–8).

Exposure to tobacco marketing that targets potential consumers by social identity can influence cigarette brand preference. Smokers report that they construct their social identities around their preferred cigarette brand and associate their preferred brand with enjoyment of smoking (9,10). When tobacco companies target marketing to LGB and other salient social identities (eg, by gender, race, or ethnicity), LGB consumers may form brand preferences on the basis of perceived congruence between their identity and the brand’s image or the impression that smoking certain cigarette brands signals LGB group membership (11). Although cigarette brand preference may differ between LGB and heterosexual smokers, little research has been conducted in this area. The tobacco industry has also targeted marketing toward other groups, such as women and racial or ethnic minority populations (12,13). However, no studies to date have examined cigarette brand preference by sexual orientation by using an intersectional approach that considers gender and race or ethnicity. Our study had 2 objectives. First, we examined relationships between sexual orientation (heterosexual, gay, lesbian, and bisexual) and cigarette brand preference in a US-based, nationally representative sample. Second, given documented smoking differences among LGB and heterosexual populations and targeted cigarette marketing to LGB populations and to women and racial or ethnic minority populations (3,4,13–15), we also examined the implications of gender and race or ethnicity for brand use by sexual orientation.

## Methods

### Data source and sample

We analyzed data from the National Survey on Drug Use and Health (NSDUH), years 2015–2017 (N = 128,740). NSDUH is an annual, publicly available, nationally representative survey that uses a complex, multistage sampling procedure to recruit participants from all 50 states and the District of Columbia (2015 was the first year that NSDUH collected sexual orientation data). NSDUH oversamples Black/African American, Hispanic/Latino, and Asian adults and participants aged 18 to 25. LGB participants are not oversampled. The survey uses a combination of computer-assisted personal interviewing and computer-assisted self-interviewing for responses to sensitive questions. The weighted response rates were 69.3% for 2015, 68.4% for 2016, and 67.1% for 2017. Our analyses were conducted in 2019–2020.

**Smoking status and cigarette brand use**. Our study included adults aged 18 or older who reported being current cigarette smokers (defined as those who reported smoking cigarettes in the past 30 days). Current smokers were asked which cigarette brand they smoked most often in the past 30 days. Because response options included more than 50 brands, we focused our analysis on participants who smoked the cigarette brands used most commonly across the 3 sexual orientation groups in our sample (heterosexual, lesbian/gay, bisexual). All 3 groups most commonly smoked these same 5 brands, a finding consistent with cigarette market share data (16).

**Sexual orientation**. To assess sexual orientation, participants were asked, “which one of the following do you consider yourself to be?” and could select one of the following options: heterosexual, lesbian or gay (one category), bisexual, and don’t know. Participants who did not provide complete sexual orientation data (refused to answer or responded “don’t know”) were excluded from our analysis (n = 279). In our analysis, we refer to males who identified as nonheterosexual as gay or bisexual and females who identified as nonheterosexual as lesbian/gay or bisexual, because, although “lesbian” is one preferred identity terminology among LGB women, LGBTQ+ (lesbian, gay, bisexual, transgender, queer, + [other sexual and gender minority identities]) advocacy groups have designated “gay” as referring to anyone of any gender who is sexually or romantically attracted to people of the same gender (17).

**Sociodemographic characteristics**. Sociodemographic characteristics included in the study were gender (in NSDUH, participants in the study were asked to report their gender and provided with the response options of male or female, age [18–25 y, 26–34 y, 35–49 y, 50–64 y, ≥65 y], annual income [intended to capture purchasing capacity for certain brands: <$20,000, $20,000–$49,999, $50,000–$74,999, ≥$75,000], and race or ethnicity [non-Hispanic Asian, non-Hispanic Black/African American, non-Hispanic Native American/Alaska Native, non-Hispanic Native Hawaiian/Other Pacific Islander, non-Hispanic White, non-Hispanic more than 1 race, Hispanic/Latino of any race]). We use the term “gender” instead of “sex” throughout to conform to the NSDUH survey construct, which uses gender to designate male or female.

### Statistical analyses

We used Stata 15 (StataCorp LLC) to conduct statistical analyses and used weighted descriptive statistics to identify the cigarette brands smokers in our sample most commonly used. We then focused our analysis on participants who had complete data for sexual orientation and were current smokers of the 5 most commonly used cigarette brands (N = 24,310). We conducted bivariate analyses of cigarette brand use by sexual orientation group stratified by sociodemographic characteristics. We then conducted weighted logistic regressions testing relationships between sexual orientation and use of each cigarette brand, controlling for sociodemographic characteristics. Finally, we conducted separate logistic regression models for each brand that contained an interaction term for either sexual orientation and gender or sexual orientation and race or ethnicity to examine the implications of gender and race or ethnicity for brand use.

## Results

More than 20% (20.4%) of the 128,740 adults who participated in the 2015–2017 NSDUH surveys reported currently smoking (n = 30,658). Approximately 20% (19.9%) of heterosexual participants, 28.1% of gay/lesbian participants, and 33.6% of bisexual participants reported current smoking (*P* < .001). The 5 brands that current adult smokers in our sample reported most commonly using were Camel (13.2%), Marlboro (52.3%), Newport (20.8%), Pall Mall (8.1%), and American Spirit (5.6%) (Natural American Spirit cigarettes were called American Spirit in the NSDUH survey). Participants in our final study sample of adult smokers (N = 24,310) (adult smokers of the 5 most commonly used brands who had complete data for sexual orientation) were 92.2% heterosexual, 2.8% lesbian/gay, and 5.0% bisexual; 56.6% were male and 43.4% were female. The racial or ethnic composition of participants in the sample was 65.7% White, 15.0% Hispanic/Latino, 12.9% Black/African American, 2.7% Asian, 2.3% more than 1 race, 1.0% Native American/Alaska Native, and 0.37% Native Hawaiian/Other Pacific Islander.

**Cigarette brand use by sexual orientation**. Marlboro cigarette use, the most commonly smoked cigarette brand in our sample, was most prevalent among heterosexual smokers (53.1% vs 46.8% of lesbian/gay smokers and 40.3% of bisexual smokers, *P* < .001) (Table 1). Newport cigarette use, the second most commonly smoked, was most prevalent among bisexual smokers (25.6% vs 20.5% of heterosexual smokers and 20.4% of lesbian/gay smokers, *P* < .001). Lesbian/gay and bisexual smokers had a higher prevalence of Camel cigarette use than heterosexual smokers (16.5% of lesbian/gay smokers and 17.2% of bisexual vs 12.9% of heterosexual smokers, *P* < .001). Lesbian/gay and bisexual smokers also had more than twice the prevalence of American Spirit use as heterosexual smokers (11.0% of lesbian/gay smokers and 11.4% of bisexual smokers vs 5.1% of heterosexual smokers, *P* < .001). Finally, heterosexual smokers had a higher prevalence of Pall Mall use than lesbian/gay or bisexual smokers (8.4% vs 5.4% lesbian/gay smokers and 5.5% bisexual smokers; *P* < .001).

**Table 1:** Characteristics of Participant Sample (N = 24,310) Stratified by Sexual Orientation and Cigarette Brand Smoked, National Survey on Drug Use and Health 2015–2017^a^

Characteristic	Camel	Marlboro	Newport	Pall Mall	American Spirit
**Heterosexual Smokers, n = 21,845**					
**Total**	3,208 (12.9)^b^	11,544 (53.1)^b^	4,710 (20.5)^b^	1,189 (8.4)^b^	1,194 (5.1)^b^
**Annual income, $**					
<20,000	648 (17.4)	2,734 (20.5)^b^	1,887 (37.5)^c^	385 (31.0)	285 (19.8)
20,000–49,999	1,187 (34.6)	4,155 (32.8)^b^	1,744 (36.1)^c^	488 (41.5)	395 (32.5)
50,000–74,999	545 (17.9)	1,769 (16.1)^b^	493 (11.5)^c^	163 (13.4)	178 (15.7)
≥75,000	828 (30.1)	2,886 (30.7)^b^	586 (14.9)^c^	153 (14.1)	336 (32.1)
**Age, y**					
18–25	1,277 (23.1)^d^	4,150 (18.1)^b^	1,755 (20.8)^b^	145 (3.9)^b^	514 (26.3)^d^
26–34	968 (30.9)^d^	2,922 (23.1)^b^	1,423 (29.0)^b^	181 (9.8)^b^	301 (25.8)^d^
35–49	756 (29.1)^d^	3,240 (31.3)^b^	1,136 (29.3)^b^	447 (27.4)^b^	274 (25.9)^d^
50–64	177 (14.4)^d^	1,031 (23.0)^b^	345 (17.9)^b^	278 (38.6)^b^	76 (17.3)^d^
≥65	30 (2.5)^d^	201 (4.5)^b^	51 (3.0)^b^	138 (20.2)^b^	29 (4.8)^d^
**Race or ethnicity**					
Asian	92 (3.2)	315 (3.5)^c^	40 (1.0)^e^	7 (1.2)	42 (4.1)
Black/African American	75 (2.9)	281 (2.7)^c^	2,227 (49.6)^e^	85 (7.6)	26 (2.5)
Hispanic/Latino	476 (16.2)	1,666 (15.9)^c^	719 (14.9)^e^	69 (6.9)	148 (12.9)
Native American/Alaska Native	75 (0.9)	419 (1.1)^c^	57 (0.6)^e^	30 (1.2)	17 (0.6)
Native Hawaiian/Other Pacific Islander	17 (0.6)	26 (0.1)^c^	30 (0.7)^e^	8 (0.4)	4 (0.2)
White	2,352 (74.2)	8,412 (74.8)^c^	1,420 (30.6)^e^	938 (80.8)	886 (76.0)
>1 race	121 (2.0)	425 (2.0)^c^	217 (2.6)^e^	52 (2.0)	71 (3.5)
**Gender**					
Male	1,799 (62.0)^b^	6,452 (58.2)^b^	2,545 (56.5)^b^	621 (53.8)	774 (64.8)^c^
Female	1,409 (38.0)^b^	5,092 (41.8)^b^	2,165 (43.5)^b^	568 (46.2)	420 (35.2)^c^
**Lesbian/Gay Smokers, n = 746**					
**Total**	129 (16.5)^b^	356 (46.8)^b^	171 (20.4)^b^	32 (5.4)^b^	58 (11.0)^b^
**Annual income, $**					
<20,000	30 (17.4)	108 (25.8)^b^	64 (38.3)^c^	12 (39.2)	10 (8.5)
20,000–49,999	49 (30.9)	112 (26.2)^b^	67 (31.4)^c^	12 (46.9)	20 (28.1)
50,000–74,999	19 (23.4)	46 (19.2)^b^	20 (15.4)^c^	6 (13.3)	8 (18.6)
≥75,000	31 (28.3)	90 (28.8)^b^	20 (15.0)^c^	2 (0.6)	20 (44.8)
**Age, y**					
18–25	59 (31.1)^d^	165 (24.4)^b^	90 (29.8)^b^	8 (9.1)^b^	23 (21.2)^d^
26–34	38 (35.8)^d^	80 (26.1)^b^	47 (33.4)^b^	4 (10.1)^b^	14 (23.2)^d^
35–49	24 (21.7)^d^	82 (27.6)^b^	27 (25.3)^b^	10 (23.3)^b^	12 (14.2)^d^
50–64	8 (11.4)^d^	24 (18.9)^b^	6 (8.2)^b^	7 (43.5)^b^	6 (25.5)^d^
≥65	0^d^	5 (3.0)^b^	1 (3.3)^b^	3 (14.1)^b^	3 (15.9)^d^
**Race or ethnicity**					
Asian	1 (3.2)	7 (1.8)^c^	0^e^	0	2 (4.0)
Black/African American	5 (6.4)	17 (4.2)^c^	83 (54.1)^e^	4 (9.1)	2 (1.8)
Hispanic/Latino	26 (23.6)	63 (17.8)^c^	39 (21.3)^e^	0	9 (17.7)
Native American/Alaska Native	6 (1.7)	12 (1.5)^c^	3 (0.3)^e^	2 (1.7)	0
Native Hawaiian/Other Pacific Islander	0	6 (1.2)^c^	2 (3.8)^e^	1 (1.4)	1 (0.2)
White	78 (59.1)	230 (71.0)^c^	33 (15.7)^e^	24 (86.6)	40 (74.2)
>1 race	13 (6.2)	21 (2.6)^c^	11 (4.8)^e^	1 (1.1)	4 (2.1)
**Gender**					
Male	68 (59.6)^b^	185 (58.9)^b^	61 (45.8)^b^	17 (69.4)	34 (67.0)^c^
Female	61 (40.4)^b^	171 (41.1)^b^	110 (54.3)^b^	15 (30.6)	24 (33.0)^c^
**Bisexual Smokers, n = 1,719**					
Total	284 (17.2)^b^	778 (40.3)^b^	424 (25.6)^b^	71 (5.5)^b^	162 (11.4)^b^
**Annual income, $**					
<20,000	80 (19.3)	281 (33.3)^b^	225 (53.5)^c^	24 (33.5)	52 (27.9)
20,000–49,999	108 (41.6)	277 (35.2)^b^	121 (28.6)^c^	27 (42.4)	60 (33.8)
50,000–74,999	50 (18.2)	91 (11.2)^b^	34 (6.1)^c^	11 (10.4)	16 (9.8)
≥75,000	46 (20.8)	129 (20.4)^b^	44 (11.9)^c^	9 (13.7)	34 (28.5)
**Age, y**					
18–25	172 (42.1)^d^	474 (43.0)^b^	233 (38.2)^b^	31 (23.4)^b^	99 (40.1)^d^
26–34	78 (36.0)^d^	194 (30.7)^b^	127 (32.9)^b^	19 (31.1)^b^	45 (41.7)^d^
35–49	32 (20.3)^d^	97 (20.8)^b^	53 (20.3)^b^	12 (11.2)^b^	17 (15.6)^d^
50–64	2 (1.6)^d^	10 (5.0)^b^	10 (7.9)^b^	8 (30.4)^b^	1 (2.7)^d^
≥65	0^d^	3 (0.6)^b^	1 (0.7)^b^	1 (4.0)^b^	0^d^
**Race or ethnicity**					
Asian	8 (4.4)	9 (1.0)^c^	3 (1.1)^e^	1 (2.7)	3 (0.7)
Black/African American	8 (4.9)	22 (2.1)^c^	181 (48.5)^e^	2 (1.8)	2 (0.9)
Hispanic/Latino	37 (18.1)	102 (11.8)^c^	68 (14.8)^e^	4 (5.0)	24 (22.6)
Native American/Alaska Native	4 (0.6)	29 (1.3)^c^	8 (0.4)^e^	0	5 (1.2)
Native Hawaiian/Other Pacific Islander	6 (0.6)	4 (0.5)^c^	0^e^	0	0
White	203 (68.6)	567 (80.9)^c^	133 (30.4)^e^	57 (86.7)	118 (70.0)
>1 race	18 (2.8)	45 (2.5)^c^	31 (4.8)^e^	7 (3.9)	10 (4.6)
**Gender**					
Male	53 (22.5)^b^	161 (24.6)^b^	41 (12.2)^b^	19 (38.2)	42 (35.6)^c^
Female	231 (77.5)^b^	617 (75.4)^b^	383 (87.8)^b^	52 (61.8)	120 (64.4)^c^

a Values are number (weighted percentage). Because of weighted percentages and rounding, some columns do not add up to 100%.

b *P* < .001, calculated by χ^2^ test.

c *P* = .001, calculated by χ^2^ test.

d *P* = .002, calculated by χ^2^ test.

e *P* = .03, calculated by χ^2^ test.

**Cigarette brand use by sexual orientation, gender, and race or ethnicity.** A greater proportion of heterosexual smokers of all brands were male than female (Table 1). Moreover, a greater proportion of lesbian/gay smokers of most brands were male than female. However, a greater proportion of lesbian/gay Newport smokers were female than male (54.3% vs 45.8%, *P* < .001). Most bisexual smokers across brands were female, and the gender difference was most pronounced among bisexual Newport smokers, of which 87.8% were female (*P* < .001).

Across sexual orientation groups, most Marlboro smokers were White or Hispanic/Latino (Table 1). Most heterosexual and bisexual Newport smokers were Black/African American or White, whereas most lesbian/gay Newport smokers were Black/African American (54.1%) or Hispanic/Latino (21.3%; *P* = .03). We found no other significant differences in brand use by race or ethnicity.

### Logistic regression

**Overall models**. In our initial models, lesbian/gay smokers had 1.7 times the odds (95% CI, 1.2–2.3) and bisexual smokers had 1.8 times the odds (95% CI, 1.4–2.1) of smoking Camel cigarettes as heterosexual smokers (Table 2). Lesbian/gay smokers had 2.8 times the odds (95% CI, 1.9–4.1) and bisexual smokers had 3.2 (95% CI, 2.5–4.1) times the odds of smoking American Spirit cigarettes as heterosexuals. Lesbian/gay smokers had 1.2 times the odds of Marlboro use as heterosexual smokers (95% CI, 1.0–1.4), and bisexual smokers had 1.7 times the odds of Newport use as heterosexual smokers (95% CI, 1.4–2.1). No significant associations were found between sexual orientation and Pall Mall use.

**Table 2:** Logistic Regression Models of Cigarette Brand Use Among Participant Sample (N = 24,310) by Sexual Orientation and Sociodemographic Characteristics, National Survey on Drug Use and Health 2015–2017^a^

Characteristic	Camel	Marlboro	Newport	Pall Mall	American Spirit
**Sexual orientation**					
Heterosexual	1[Reference]	1[Reference]	1[Reference]	1[Reference]	1[Reference]
Lesbian/gay	1.7 (1.2–2.3)^b^	1.2 (1.0–1.4)^c^	1.2 (0.9–1.5)	0.9 (0.6–1.5)	2.8 (1.9–4.1)^d^
Bisexual	1.8 (1.5–2.2)^d^	1.1 (1.0–1.3)	1.7 (1.4–2.1)^d^	1.5 (1.0–2.2)	3.2 (2.5–4.1)^d^
**Annual income, $**					
<20,000	1.5 (1.3–1.7)^d^	2.1 (2.0–2.3)^d^	4.0 (3.5–4.6)^d^	7.9 (6.0–10.5)^d^	1.5 (1.2–1.9)^e^
20,000–49,999	1.7 (1.5–1.9)^d^	1.7 (1.6–1.9)^d^	2.4 (2.1–2.7)^d^	5.1 (4.0–6.6)^d^	1.4 (1.1–1.7)^c^
50,000–74,999	1.5 (1.3–1.7)^d^	1.4 (1.3–1.5)^d^	1.5 (1.3–1.8)^d^	2.6 (1.9–3.4)^d^	1.1 (0.9–1.5)
≥75,000	1[Reference]	1[Reference]	1[Reference]	1[Reference]	1[Reference]
**Age, y**					
18–25	16.4 (10.1–26.7)^d^	7.4 (6.2–8.9)^d^	8.2 (5.7–11.8)^d^	0.3 (0.2–0.5)^d^	7.5 (4.7–12.0)^d^
26–34	19.5 (11.7–32.4)^d^	8.8 (7.4–10.5)^d^	12.0 (8.4–17.0)^d^	0.8 (0.7–1.1)	7.1 (4.4–11.5)^d^
35–49	11.8 (7.2–19.4)^d^	7.7 (6.3–9.2)^d^	8.2 (5.7–11.8)^d^	1.6 (1.3–2.1)^d^	4.4 (2.7–7.1)^d^
50–64	5.1 (2.9–8.8)^d^	4.8 (3.9–5.8)^d^	4.6 (3.1–6.6)^d^	2.0 (1.5–2.6)^d^	2.7 (1.6–4.6)^f^
≥65	1[Reference]	1[Reference]	1[Reference]	1[Reference]	1[Reference]
**Race or ethnicity**					
Asian	0.4 (0.3–0.6)^d^	0.5 (0.4–0.6)^d^	0.3 (0.2–0.5)^d^	0.3 (0.1–0.5)^f^	0.5 (0.3–0.9)^c^
Black/African American	0.2 (0.1–0.2)^d^	0.1 (0.1–0.2)^d^	7.2 (6.5–8.0)^d^	0.4 (0.3–0.5)^d^	0.1 (0.1–0.2)^d^
Hispanic/Latino	0.7 (0.6–0.7)^d^	0.6 (0.6–0.7)^d^	1.4 (1.2–1.6)^c^	0.3 (0.2–0.4)^d^	0.6 (0.4–0.7)^d^
Native American/Alaska Native	1.2 (0.9–1.6)	1.4 (1.1–1.6)^b^	1.5 (0.9–2.4)	1.1 (0.6–2.1)	0.8 (0.4–1.5)
Native Hawaiian/Other Pacific Islander	1.2 (0.4–3.5)	0.3 (0.2–0.5)^d^	3.7 (2.2–6.3)^d^	0.7 (0.2–2.0)	0.4 (0.1–1.3)
White	1[Reference]	1[Reference]	1[Reference]	1[Reference]	1[Reference]
>1 race	0.9 (0.7–1.2)	0.9 (0.7–1.1)	2.7 (2.1–3.5)^d^	0.8 (0.5–1.2)	1.4 (1.0–2.0)
**Gender**					
Male	1[Reference]	1[Reference]	1[Reference]	1[Reference]	1[Reference]
Female	0.6 (0.6–0.7)^d^	0.7 (0.70–0.74)^d^	0.7 (0.6–0.8)^d^	0.7 (0.6–0.8)^d^	0.5 (0.5–0.6)^d^

a Values are odds ratio (95% CI). Each model represents a separate and independent analysis, but models were combined for simultaneous viewing.

b *P* = .003, calculated by *t* test.

c *P* < .05, calculated by *t* test.

d *P* < .001, calculated by *t* test.

e *P* = .002, calculated by *t* test.

f *P* = .001, calculated by *t* test.

Females had significantly lower odds of use of all brands than males. Native American/Alaska Native smokers had higher odds of Marlboro use than White smokers (OR = 1.4; 95% CI, 1.1–1.6) (Table 2). Black/African American smokers (OR = 7.2; 95% CI, 6.5–8.0), smokers who identified as more than 1 race (OR = 2.7; 95% CI, 2.1–3.5), Native Hawaiian/Pacific Islander (OR = 3.7; 95% CI, 2.2–6.3), and Hispanic/Latino smokers (OR = 1.4; 95% CI, 1.2–1.6) had higher odds of Newport use than White smokers. We found no other significant positive associations between race or ethnicity and cigarette brands used.

Income and age were also significant predictors of use across brands. Younger age groups (18–25 y, 26–34 y) were more likely than the oldest age group (≥65 y) to use all brands except for Pall Mall (younger groups were less likely to use this brand). Lower annual income groups (<$20,000, $20,000–$49,999 income) were more likely than the highest income group (≥$75,000) to use all brands.

**Interaction analysis: sexual orientation and gender**. Significant interactions between sexual orientation and gender were found for Camel, Marlboro, and Newport smokers (Table 3). We found a significant positive association between bisexual female identity and both Camel cigarette use (OR = 1.8; 95% CI, 1.3–2.7) and Marlboro use (OR = 1.6; 95% CI, 1.1–2.2) vs bisexual male smokers but did not find the same association among lesbian/gay female smokers. We also found a significant positive association in use of Newport cigarettes among both bisexual female smokers (OR = 3.4; 95% CI, 2.0–5.7 vs bisexual male) and lesbian/gay female smokers (OR = 1.8; 95% CI, 1.1–3.2 vs gay male smokers), which was pronounced among bisexual females.

**Table 3:** Logistic Regression Models Among Participant Sample (N = 24,310) in Relation to Cigarette Choice, by Sexual Orientation and Sociodemographic Characteristics, National Survey on Drug Use and Health 2015–2017^a^

Characteristic	Interaction Effects of Sexual Orientation and Gender			Interaction Effects of Sexual Orientation and Race or Ethnicity	
	Camel	Marlboro	Newport	Marlboro	Newport
**Sexual orientation**					
Heterosexual	1[Reference]	1[Reference]	1[Reference]	1[Reference]	1[Reference]
Lesbian/gay	1.5 (0.9–2.4)	1.1 (0.9–1.4)	0.9 (0.6–1.3)	1.1 (0.9–1.4)	0.6 (0.4–1.1)
Bisexual	1.2 (0.8–1.7)	0.8 (0.6–1.1)	0.7 (0.4–1.1)	1.3 (1.1–1.5)^b^	1.6 (1.3–2.0)^c^
**Annual income, $**					
<20,000	1.5 (1.3–1.7)^c^	2.1 (2.0–2.3)^c^	4.0 (3.5–4.6)^c^	2.1 (2.0–2.3)^c^	4.0 (3.5–4.6)^c^
20,000–49,999	1.7 (1.5–1.9)^c^	1.7 (1.6–1.9)^c^	2.4 (2.1–2.7)^c^	1.7 (1.6–1.9)^c^	2.4 (2.1–2.7)^c^
50,000–74,999	1.5 (1.3–1.7)^c^	1.4 (1.3–1.5)^c^	1.6 (1.3–1.8)^c^	1.4 (1.3–1.5)^c^	1.5 (1.3–1.8)^c^
≥75,000	1[Reference]	1[Reference]	1[Reference]	1[Reference]	1[Reference]
**Age, y**					
18–25	16.3 (10.0–26.5)^c^	7.4 (6.2–8.9)^c^	8.0 (5.6–11.6)^c^	7.4 (6.2–8.9)^c^	8.2 (5.7–11.9)^c^
26–34	19.3 (11.6–32.2)^c^	8.8 (7.4–10.5)^c^	11.8 (8.3–16.8)^c^	8.8 (7.4–10.5)^c^	12.1 (8.5–17.2)^c^
35–49	11.7 (7.1–19.3)^c^	7.6 (6.3–9.2)^c^	8.1 (5.6–11.7)^c^	7.6 (6.3–9.2)^c^	8.2 (5.7–11.9)^c^
50–64	5.1 (2.9–8.8)^c^	4.8 (3.9–5.8)^c^	4.5 (3.1–6.6)^c^	4.8 (3.9–5.8)^c^	4.6 (3.1–6.7)^c^
≥65	1[Reference]	1[Reference]	1[Reference]	1[Reference]	1[Reference]
**Race/ethnicity**					
Asian	0.4 (0.3–0.6)^c^	0.5 (0.4–0.6)^c^	0.3 (0.2–0.5)^c^	0.5 (0.4–0.6)^c^	0.3 (0.2–0.5)^c^
Black/African American	0.2 (0.1–0.2)^c^	0.1 (0.1–0.2)^c^	7.2 (6.5–8.0)^c^	0.1 (0.1–0.2)^c^	7.1 (6.3–7.9)^c^
Hispanic/Latino	0.7 (0.6–0.7)^c^	0.6 (0.6–0.7)^c^	1.4 (1.2–1.6)^c^	0.6 (0.6–0.7)^c^	1.3 (1.1–1.6)^d^
Native American/Alaska Native	1.2 (0.9–1.6)	1.4 (1.1–1.6)^e^	1.5 (0.9–2.4)	1.3 (1.1–1.7)^f^	1.5 (0.9–2.5)
Native Hawaiian/Other Pacific Islander	1.3 (0.4–3.6)	0.3 (0.2–0.5)^c^	3.8 (2.2–6.4)^c^	0.2 (0.1–0.4)^c^	3.4 (1.9–5.9)^c^
White	1[Reference]	1[Reference]	1[Reference]	1[Reference]	1[Reference]
>1 race	0.9 (0.7–1.2)	0.9 (0.7–1.1)	2.7 (2.1–3.5)^c^	0.9 (0.7–1.2)	2.6 (2.0–3.5)^c^
**Gender**					
Male	1[Reference]	1[Reference]	1[Reference]	1[Reference]	1[Reference]
Female	0.6 (0.5–0.7)^c^	0.7 (0.6–0.7)^c^	0.7 (0.6–0.7)^c^	0.7 (0.65–0.7)^c^	0.7 (0.6–0.8)^c^
**Sexual orientation and gender** g					
Gay male	1[Reference]	1[Reference]	1[Reference]	1[Reference]	1[Reference]
Lesbian/gay female	1.5 (0.7–2.8)	1.3 (0.9–1.9)	1.8 (1.1–3.2)^f^	—	—
Bisexual male	1[Reference]	1[Reference]	1[Reference]	1[Reference]	1[Reference]
Bisexual female	1.8 (1.3–2.7)^h^	1.6 (1.1–2.2)^f^	3.4 (2.0–5.7)^c^	—	—
**Sexual orientation and race or ethnicity** i					
Lesbian/gay Asian	—	—	—	0.7 (0.2–2.1)	—
Lesbian/gay Black/ African American	—	—	—	1.4 (0.7–2.7)	1.9 (0.8–4.4)
Lesbian/gay Hispanic/Latino	—	—	—	1.2 (0.8–1.9)	3.2 (1.5–6.5)^h^
Lesbian/gay Native American/Alaska Native	—	—	—	1.7 (0.6–4.8)	0.8 (0.1–5.7)
Lesbian/gay Native Hawaiian/Other Pacific Islander	—	—	—	7.5 (1.4–39.9)^f^	7.7 (1.2–47.9)^f^
Lesbian/gay White	—	—	—	Reference	Reference
Lesbian/gay >1 race	—	—	—	0.9 (0.5–1.7)	2.6 (0.5–15.1)
Bisexual Asian	—	—	—	0.3 (0.1–0.8)^f^	1.4 (0.3–6.3)
Bisexual Black/African American	—	—	—	0.7 (0.4–1.3)	1.2 (0.8–1.6)
Bisexual Hispanic/Latino	—	—	—	0.7 (0.5–1.0)	1.2 (0.7–1.8)
Bisexual Native American/Alaska Native	—	—	—	1.0 (0.4–2.2)	0.7 (0.2–2.2)
Bisexual Native Hawaiian/Other Pacific Islander	—	—	—	7.9 (2.0–31.4)^b^	__
Bisexual White	—	—	—	Reference	—
Bisexual >1 race	—	—	—	0.5 (0.3–0.9)^f^	1.0 (0.6–1.8)

Abbreviation: — , not included in model because there were no participants in this category.

a Tests the interaction effects of sexual orientation and gender and sexual orientation and race or ethnicity (brands included represent brands for which significant interactions for gender, race, or ethnicity were found). Values are odds ratio (95% CI). Each model represents a separate and independent analysis, but models were combined for simultaneous viewing.

b *P* = .004, calculated by *t* test.

c *P* < .001, calculated by *t* test.

d *P* = .001, calculated by *t* test.

e *P* = .003, calculated by *t* test.

f *P* < .05, calculated by *t* test.

g Base categories of heterosexual and gender omitted from model. Analyses within lesbian/gay and bisexual groups are shown.

h *P* = .002, calculated by *t* test.

i Base categories of heterosexual and race or ethnicity omitted from model. Analyses within lesbian/gay and bisexual groups are shown.

**Interaction analysis: sexual orientation and race or ethnicity**. Significant interactions between sexual orientation and race or ethnicity were found for Marlboro and Newport use (Table 3). We found a significant positive association between smokers identifying as lesbian/gay Native Hawaiian/Other Pacific Islander (vs lesbian/gay White smokers) for both Marlboro and Newport use (Marlboro, OR = 7.5; 95% CI, 1.4–39.9; Newport OR = 7.7; 95% CI, 1.2–47.9). Additionally, we found a significant positive association between bisexual identity and Native Hawaiian/Other Pacific Islander ethnicity (vs bisexual White smokers) and Marlboro use (OR = 7.9; 95% CI, 2.0–31.4). We also found a significant positive association between lesbian/gay Hispanic smoker identity (vs lesbian/gay White smoker identity) (OR = 3.2; 95% CI, 1.5–6.5) and Newport use. Finally, significant negative associations were found for use of Marlboro cigarettes between both smokers of bisexual identity and Asian race (OR = 0.3; 95% CI, 0.1–0.8) and smokers of bisexual identity who identified as more than 1 race (OR = 0.5; 95% CI, 0.3–0.9), implying that bisexual Asian smokers and bisexual smokers of more than 1 race were less likely to use Marlboro cigarettes than bisexual White smokers.

## Discussion

Our study provides an in-depth analysis of cigarette brand use by sexual orientation, examining the role of gender and race or ethnicity in brand use among LGB smokers. US adult smokers who identified as lesbian/gay or bisexual were more likely to smoke certain commonly used cigarette brands than heterosexuals (Camel, Marlboro, Newport, and American Spirit). Gender (conceptualized in the NSDUH survey as male/female) and race or ethnicity also had implications for the use of certain cigarette brands among LGB smokers.

Several factors may have contributed to the increased likelihood of Camel use among LGB smokers versus heterosexual smokers, one potential influence being targeted marketing. For example, RJ Reynolds’s Project SCUM (4) was one of the first known LGB-targeted cigarette brand marketing campaigns, which may have contributed to Camel use among LGB smokers in our sample. We also found a positive association between bisexual female identity and Camel use. Specialty Camel brands targeted to young women, such as Camel No. 9, may have contributed to this association (18). However, we did not find a significant interaction between being female and identifying as lesbian/gay. Camel brand preference among bisexual females may therefore be influenced by factors that transcend targeted marketing to the general population of women.

Gay/lesbian smokers had slightly higher odds of Marlboro use than heterosexual smokers, and we found a significant interaction between bisexual identity and gender for Marlboro use, wherein bisexual female smokers were more likely to use Marlboro cigarettes than bisexual male smokers. However, we did not find increased odds among bisexual male or lesbian/gay female smokers. A history of overt targeted marketing of Marlboro cigarettes to gay men (19) and implicit targeted marketing to LGB women (20) suggests a need for further studies of gender-targeted Marlboro advertisements and other contributors to Marlboro use among LGB smokers.

We also found associations between the interaction of both lesbian/gay and bisexual Native Hawaiian/Other Pacific Islander identity and Marlboro use, but the CIs of both findings were wide (possibly because of small cell sizes) and should be interpreted with caution. Although little evidence exists of targeted Marlboro cigarette marketing to Native Hawaiian/Other Pacific Islander populations, robust evidence has shown that several tobacco companies have targeted those populations since the 1980s through advertising, commercial relationship building, and sponsorships (12). However, further research examining factors contributing to Marlboro brand use among LGB Native Hawaiian/Other Pacific Islander populations is needed.

Identifying as bisexual was associated with increased odds of using Newport cigarettes, which are primarily menthol cigarettes (21). This reflects prior findings that bisexual smokers are more likely than heterosexual smokers to smoke menthol cigarettes (22). Although few studies have examined Newport advertising that targets LGBTQ+ populations, one review of Newport marketing practices found that the Newport brand has historically used nightlife imagery to sell its products (23). This may have had implications for LGB Newport use in this study, because LGBTQ+ nightlife venues have long been known as important social spaces for relationship building in LGBTQ+ communities (24). Moreover, we found that Newport use was more prevalent among young bisexual smokers, who may engage in nightlife activities more frequently than older bisexual smokers. This result was not found for lesbian/gay smokers, and research is needed on factors influencing Newport brand preference across LGB subgroups.

Gender also played a part in Newport use by sexual orientation, and female lesbian/gay and female bisexual smokers were more likely to use Newport than male gay or bisexual smokers. This mirrors studies showing that LGB female smokers have high rates of menthol cigarette use (22). This finding emerged despite one study showing that female menthol smokers were more likely than their counterparts who smoked nonmenthol cigarettes to perceive their cigarettes as harmful (25). This finding may be partially related to targeted menthol cigarette marketing to women (26), but no research to date has examined factors influencing Newport cigarette use among LGB females.

Race or ethnicity also had implications for Newport use in our overall and interaction models. Black/African American race or Hispanic/Latino ethnicity was associated with Newport use in the overall models, which reflects previous studies (27). Moreover, having a racial or ethnic identity of Native Hawaiian/Pacific Islander or more than 1 race was associated with Newport use. We also found a significant positive association between the interaction of lesbian/gay identity and Hispanic/Latino ethnicity and Newport cigarette use. Newport’s marketing, which may appeal to LGBTQ+ populations (23) and documented targeted marketing of Newport and other menthol cigarette brands to Hispanic/Latino populations (12,13,28) may have possibly acted as intersectional targeted marketing that had implications for Newport use in our study. However, although Newport and other menthol cigarette brands have heavily targeted Black/African American populations (12,13), we did not find significant interactions between Black/African American race and sexual orientation. Finally, we found a significant positive association between lesbian/gay identity and Native Hawaiian/Other Pacific Islander race or ethnicity and Newport use, which may be related to the aforementioned targeted marketing efforts. However, as was found with Marlboro use, the CIs of this finding were very wide and should be interpreted cautiously.

Lesbian/gay and bisexual smokers had approximately 3 times the odds of American Spirit use as heterosexual smokers. This may have been influenced by targeted marketing in LGB publications (5). Although a previous study found higher odds of American Spirit use among LGB smokers than heterosexual smokers, the odds of use in our sample exceeded those found previously (29). This may be due to the more recent years of data collection in our study (2015–2017) than in that previous study (2013–2014), given increases in American Spirit cigarette use over time (16). The increase in American Spirit use in recent years may be due to evidenced receptivity among smokers to American Spirit brand marketing that characterizes the cigarettes as “natural” and “additive free,” which contributes to perceptions of lower risk from smoking American Spirit cigarettes (29). Although one study found no significant differences in responses to this type of “health-oriented” cigarette marketing by sexual orientation (29), further study is needed about aspects of American Spirit marketing that may appeal to LGB smokers.

Our findings lay the groundwork for further studies of cigarette brand preference among LGB populations. Furthermore, our study demonstrates the importance of studying LGB population subgroups, because we found differences not only in brand use between LGB and heterosexual smokers, but also between lesbian/gay and bisexual smokers. By also examining the associations between gender and race or ethnicity and brand use in LGB smokers, our study contributes an intersectional examination of cigarette brand use. Finally, understanding demographic correlates of brand preference in populations experiencing tobacco-related health disparities may inform tobacco regulation at the federal, state, and local levels pertaining to targeted marketing of specific brands and associated effects on public health.

Our study had limitations. First, NSDUH did not assess the origins of use of certain brands among LGB populations (eg, tobacco marketing). Additionally, the sexual orientation measure in NSDUH did not account for all emerging sexual identities or all terminologies. Furthermore, NSDUH inquired about participant sex (male/female) rather than gender identity, despite labeling the variable/construct “gender.” Also, because the gender variable was operationalized in binary terms, brand use in noncisgender (people whose gender identities do not correspond with their birth sex) populations cannot be determined from this survey. Although NSDUH collected data from several racial or ethnic groups, analyses were limited by small sample sizes in some groups. However, collapsing or excluding certain racial or ethnic groups from analysis may compound health disparities and erase the experiences of individuals in certain groups. Our study focused on intersections between sexual orientation, gender, race or ethnicity, and cigarette brand use. Future studies could consider examining other characteristics that may be included in marketing efforts, such as age and income. However, in our study, we did not find noteworthy variations across brands; younger age groups were more likely than older groups to use almost all brands, and low-income smokers were more likely than high-income smokers to use all brands. Additionally, because the NSDUH measure of brand use allowed participants to choose only one brand, we were not able to examine multibrand use among participants. Therefore, given the numerous cigarette brands available, further research on multibrand use and potential overlaps in predictors of the use of certain brands (eg, use of menthol cigarettes from multiple brands, use of multiple brands with similar marketing strategies) is needed. Finally, we studied cigarette brand use, and given the increased use of e-cigarettes in the US in recent years, future studies could include brand preference for e-cigarettes (NSDUH does not contain questions about e-cigarette use).

LGB smokers may be more likely than heterosexual smokers to smoke some commonly used cigarette brands. Moreover, gender and race or ethnicity may have implications for brand preference among LGB smokers. Targeted marketing by sexual orientation, gender, and race or ethnicity is an important contributor to brand use. Future research could consider examining the implications of targeted marketing and other potential contributors to brand use among LGBTQ+ smokers by using an intersectional approach that includes a wide variety of sexual orientations, gender identities, and races and ethnicities.

## References

[1] US Department of Health and Human Services. The health consequences of smoking — 50 years of progress. A report of the Surgeon General. 2014. https://www.cdc.gov/tobacco/campaign/tips/resources/data/cigarette-smoking-in-united-states.html#:~:text=In%202019%2C%20an%20estimated%2014.0,every%20day%20or%20some%20days. Accessed February 3, 2021.

[2] Centers for Disease Control and Prevention. Burden of tobacco use in the U.S. 2019. https://www.cdc.gov/tobacco/campaign/tips/resources/data/cigarette-smoking-in-united-states.html. Accessed August 24, 2021.

[3] Stevens P , Carlson LM , Hinman JM . An analysis of tobacco industry marketing to lesbian, gay, bisexual, and transgender (LGBT) populations: strategies for mainstream tobacco control and prevention. Health Promot Pract 2004;5(3 Suppl):129S–34S. 10.1177/1524839904264617 15231106

[4] Reynolds RJ . Project SCUM. 1995. Truth Tobacco Industry Documents. https://www.industrydocuments.ucsf.edu/tobacco/docs/#id=sfck0098. Accessed August 24, 2021.

[5] Trinkets and Trash. Trinkets and trash: artifacts of the tobacco epidemic. http://www.trinketsandtrash.org. Accessed November 10, 2020.

[6] Emory K , Buchting FO , Trinidad DR , Vera L , Emery SL . Lesbian, gay, bisexual, and transgender (LGBT) view it differently than non-LGBT: exposure to tobacco-related couponing, e-cigarette advertisements, and anti-tobacco messages on social and traditional media. Nicotine Tob Res 2019;21(4):513–22. 10.1093/ntr/nty049 29546337 PMC6472708

[7] Smith EA , Thomson K , Offen N , Malone RE . “If you know you exist, it’s just marketing poison”: meanings of tobacco industry targeting in the lesbian, gay, bisexual, and transgender community. Am J Public Health 2008;98(6):996–1003. 10.2105/AJPH.2007.118174 18445800 PMC2377293

[8] Lee JGL , Blanchflower TM , O’Brien KF , Cofie LE , Gregory KR , Averett PE . Assessing the potential impact of cigarette packs designed for lesbian, gay, bisexual, and transgender adults: a randomized experiment to inform U.S. regulation, 2018. Health Promot Pract 2020;21(1_suppl):157S–64S. 10.1177/1524839919879923 31908205 PMC6951441

[9] de Chernatony L . From brand vision to brand evaluation: strategically building and sustaining brands. Oxford (UK): Butterworth-Heinemann; 2001.

[10] Johnson SE , Coleman BN , Schmitt CL . It’s complicated: examining smokers’ relationships with their cigarette brands. Psychol Addict Behav 2016;30(8):887–94. 10.1037/adb0000225 27831717 PMC5702884

[11] Sirgy MJ , Lee DJ , Johar JS , Tidwell J . Effect of self-congruity with sponsorship on brand loyalty. J Bus Res 2008;61(10):1091–7. 10.1016/j.jbusres.2007.09.022

[12] National Cancer Institute. The role of the media in promoting and reducing tobacco use. Davis R, Gilpin E, Loken B, Viswanath K, Wakefield M, editors. Washington (DC): National Cancer Institute; 2008. https://cancercontrol.cancer.gov/brp/tcrb/monographs/19/m19_complete.pdf. Accessed October 15, 2020.

[13] Mills SD , Henriksen L , Golden SD , Kurtzman R , Kong AY , Queen TL , Disparities in retail marketing for menthol cigarettes in the United States, 2015. Health Place 2018;53:62–70. 10.1016/j.healthplace.2018.06.011 30055469 PMC6161357

[14] Dilley JA , Spigner C , Boysun MJ , Dent CW , Pizacani BA . Does tobacco industry marketing excessively impact lesbian, gay and bisexual communities? Tob Control 2008;17(6):385–90. 10.1136/tc.2007.024216 18723561

[15] Sharma A , Fix BV , Delnevo C , Cummings KM , O’Connor RJ . Trends in market share of leading cigarette brands in the USA: National Survey on Drug Use and Health 2002–2013. BMJ Open 2016;6(1):e008813. 10.1136/bmjopen-2015-008813 26826144 PMC4735173

[16] University of California Davis Health. LGBTQ+ glossary. 2016. https://health.ucdavis.edu/diversity-inclusion/LGBTQI/LGBTQ-Plus.html. Accessed January 11, 2021.

[17] Pierce JP , Messer K , James LE , White MM , Kealey S , Vallone DM , Camel No. 9 cigarette-marketing campaign targeted young teenage girls. Pediatrics 2010;125(4):619–26. 10.1542/peds.2009-0607 20231181

[18] Goebel K . Lesbian and gays face tobacco targeting. Tob Control 1994;3(1):65–7. 10.1136/tc.3.1.65

[19] Ling PM , Glantz SA . Using tobacco-industry marketing research to design more effective tobacco-control campaigns. JAMA 2002;287(22):2983–9. 10.1001/jama.287.22.2983 12052128

[20] Reynolds RJ . What we make. 2015. https://www.rjrt.com/transforming-tobacco/what-we-make/. Accessed February 1, 2020.

[21] Fallin A , Goodin AJ , King BA . Menthol cigarette smoking among lesbian, gay, bisexual, and transgender adults. Am J Prev Med 2015;48(1):93–7. 10.1016/j.amepre.2014.07.044 25245795 PMC4454462

[22] Balbach ED , Gasior RJ , Barbeau EMRJ . R.J. Reynolds’ targeting of African Americans: 1988-2000. Am J Public Health 2003;93(5):822–7. 10.2105/AJPH.93.5.822 12721151 PMC1447846

[23] Leibel K , Lee JGL , Goldstein AO , Ranney LM . Barring intervention? Lesbian and gay bars as an underutilized venue for tobacco interventions. Nicotine Tob Res 2011;13(7):507–11. 10.1093/ntr/ntr065 21498874

[24] Cohn AM , Rose SW , Ilakkuvan V , Gray T , Curry L , Villanti AC , Harm perceptions of menthol and nonmenthol cigarettes differ by brand, race/ethnicity, and gender in US adult smokers: results from PATH wave 1. Nicotine Tob Res 2019;21(4):439–49. 10.1093/ntr/ntx277 29385527

[25] Lawrence D , Rose A , Fagan P , Moolchan ET , Gibson JT , Backinger CL . National patterns and correlates of mentholated cigarette use in the United States. Addiction 2010;105(Suppl 1):13–31. 10.1111/j.1360-0443.2010.03203.x 21059133

[26] Rath JM , Villanti AC , Williams VF , Richardson A , Pearson JL , Vallone DM . Correlates of current menthol cigarette and flavored other tobacco product use among U.S. young adults. Addict Behav 2016;62:35–41. 10.1016/j.addbeh.2016.05.021 27310032 PMC4955768

[27] Fernandez S , Hickman N , Klonoff EA , Landrine H , Kashima K , Parekh B , Cigarette advertising in magazines for Latinas, White women, and men, 1998–2002: a preliminary investigation. J Community Health 2005;30(2):141–51. 10.1007/s10900-004-1097-5 15810566

[28] Pearson JL , Johnson A , Villanti A , Glasser AM , Collins L , Cohn A , Misperceptions of harm among Natural American Spirit smokers: results from wave 1 of the Population Assessment of Tobacco and Health (PATH) study (2013-2014). Tob Control 2017;26(e1):e61–7. 10.1136/tobaccocontrol-2016-053265 27924008

[29] Lee SJ , Sanders-Jackson A , Fallin-Bennett A , Tan ASL . Comparing the effects of organic, natural, and no additives labels on tobacco packaging between Lesbian, Gay, Bisexual (LGB) and heterosexual smokers. Addict Behav 2019;91:175–9. 10.1016/j.addbeh.2018.08.002 30119938

